# Prognostic significance of multiple kallikreins in high-grade astrocytoma

**DOI:** 10.1186/s12885-015-1566-5

**Published:** 2015-08-01

**Authors:** Kristen L. Drucker, Caterina Gianinni, Paul A. Decker, Eleftherios P. Diamandis, Isobel A. Scarisbrick

**Affiliations:** 1 Department of Physical Medicine and Rehabilitation, Mayo Medical and Graduate School, Mayo Clinic Rochester, 200 First Street SW, Rochester, MN 55905 USA; 2 Department of Laboratory Medicine and Pathology, Mayo Medical and Graduate School, Mayo Clinic Rochester, 200 First Street SW, Rochester, MN 55905 USA; 3 Biomedical Statistics and Informatics, Mayo Medical and Graduate School, Mayo Clinic Rochester, 200 First Street SW, Rochester, MN 55905 USA; 4 Department of Pathology and Laboratory Medicine, Mount Sinai Hospital, 600 University Ave, Toronto, ON M5T 3 L9 Canada; 5 Department of Physiology and Biomedical Engineering, Mayo Medical and Graduate School, Mayo Clinic Rochester, 200 First Street SW, Rochester, MN 55905 USA

**Keywords:** Glioblastoma, Kallikrein, Prognosis

## Abstract

**Background:**

Kallikreins have clinical value as prognostic markers in a subset of malignancies examined to date, including kallikrein 3 (prostate specific antigen) in prostate cancer. We previously demonstrated that kallikrein 6 is expressed at higher levels in grade IV compared to grade III astrocytoma and is associated with reduced survival of GBM patients.

**Methods:**

In this study we determined KLK1, KLK6, KLK7, KLK8, KLK9 and KLK10 protein expression in two independent tissue microarrays containing 60 grade IV and 8 grade III astrocytoma samples. Scores for staining intensity, percent of tumor stained and immunoreactivity scores (IR, product of intensity and percent) were determined and analyzed for correlation with patient survival.

**Results:**

Grade IV glioma was associated with higher levels of kallikrein-immunostaining compared to grade III specimens. Univariable Cox proportional hazards regression analysis demonstrated that elevated KLK6- or KLK7-IR was associated with poor patient prognosis. In addition, an increased percent of tumor immunoreactive for KLK6 or KLK9 was associated with decreased survival in grade IV patients. Kaplan-Meier survival analysis indicated that patients with KLK6-IR < 10, KLK6 percent tumor core stained < 3, or KLK7-IR < 9 had a significantly improved survival. Multivariable analysis indicated that the significance of these parameters was maintained even after adjusting for gender and performance score.

**Conclusions:**

These data suggest that elevations in glioblastoma KLK6, KLK7 and KLK9 protein have utility as prognostic markers of patient survival.

**Electronic supplementary material:**

The online version of this article (doi:10.1186/s12885-015-1566-5) contains supplementary material, which is available to authorized users.

## Background

Expression levels of select kallikreins (KLK) are proposed or already used as biomarkers in human malignancies, including prostate, ovarian or breast cancers. KLKs are a family of secreted serine proteases, consisting of 15 genes located in a contiguous cluster on chromosome 19q13.4 [1]. KLKs participate in trypsin- or chymotrypsin-like protein cleavage, leading to extracellular matrix degradation and tissue remodeling, activation or inactivation of other protease family members, or in some cases, activation of protease activated receptors (PARs) to elicit intracellular signaling and defined cellular responses [2]. For example, elevated levels of KLK6 are associated with higher grade, later stage and serous histotype ovarian cancer, all of which are associated with an unfavorable prognosis [3]. KLK3 (prostate specific antigen (PSA)) serves as a well-recognized serum biomarker for prostate cancer [4]. Providing the rationale for the current study, we recently demonstrated that elevated levels of KLK6 are associated with high-grade glioma and poor patient survival [5]. Very little is known regarding the potential prognostic significance of other kallikrein family members in glial tumors and here we examined the association of 6 kallikreins with GBM grade and patient survival.

The location of kallikrein family members on human chromosome 19q makes them of particular interest in glioma, given the frequency of copy number variations in glioma patient tumors [6, 7]. Whole arm loss of chromosome 19q has been linked to better survival in oligodendroglioma, although smaller deletions have not been shown to have the same survival benefit [8, 9]. Conversely, gain of chromosome 19 in glioblastoma has been correlated with a poor prognosis [10], an effect attributed to radiation resistance [11]. In this regard it is of interest that not only are levels of KLK6 significantly elevated in high-grade glioma (glioblastoma multiforme (GBM), grade IV astrocytoma) and associated with poor patient survival, but in addition KLK6 promotes the resistance of glioma cells to a wide variety of cell death-inducing agents, including staurosporine, cisplatin, radiation and temozolomide [5]. The potential pathophysiological significance of KLK6 to glioma appears to extend to lower grade tumors as well, since patients with mixed intracranial tumors positive for KLK6 expression also have unfavorable prognoses compared to those lacking expression [12]. Interestingly, patients with KLK7 positive tumors also survived for shorter intervals post-surgery relative to patients in which no KLK7 expression was detected [13]. By contrast, tumor KLK8 RNA expression in the same patient cohort was not associated with survival [13].

Given the established prognostic significance of KLK6 to GBM patient survival, taken with the association of other KLKs with a variety of CNS tumor types, we made a comprehensive examination of five additional kallikreins in the patient cohort previously utilized to determine the prognostic significance of KLK6. Like KLK6, higher levels of KLK1, KLK7, KLK8, KLK9 and KLK10 were all found to be associated with higher astrocytoma grade. In addition, high tumor levels of KLK7-IR, were, like KLK6, found to be associated with reduced patient survival. These findings suggest that multiple kallikreins are positioned to play roles in the pathophysiology of high-grade glioma and that future studies are needed to determine their biological actions including roles in directing therapeutic response.

## Methods

### Clinical samples

The tissue microarrays containing grade III and grade IV astrocytoma specimens utilized in this study were previously described in detail [5]. Briefly, surgically resected astrocytoma samples were formalin-fixed and paraffin-embedded prior to examination by a neuropathologist (CG) to determine grade III or grade IV status based on WHO criteria. Astrocytoma samples were arranged across two tissue independent microarrays in triplicate. One array contained 38 grade IV astrocytomas and the second 22 grade IV and 8 grade III astrocytomas. Five-micron paraffin sections were cut for immunohistochemical localization of kallikreins. Patient demographics are provided in Additional file 1: Table S1, including age at surgery, gender and Eastern Cooperative Oncology Group (ECOG) performance score. Mayo Clinic Institutional Review Board approved the use of all human materials utilized in this study. Informed written consent was obtained prior to donation of tissue.

### Immunohistochemical analysis

To determine the expression of kallikrein proteins in grade III and IV astrocytoma, tissue microarrays were immunostained using antibodies specific to KLK1, KLK6, KLK7, KLK8, KLK9, or KLK10 [14–16]. Deparaffinized sections were treated with 0.3 % hydrogen peroxide in methanol, followed by rehydration in graded ethyl alcohols. Primary antibodies for each kallikrein were applied for 18 h at 4 C. KLK1 immunoreactivity was detected using a mouse monoclonal antibody M01-H00003816 (Novus Biologicals, Littleton, CO). KLK6 was detected using a KLK6-specific monoclonal antibody (MSP-3-3) [14–16]. KLK7 [17, 18], KLK8 [19] and KLK10 [17] were detected with previously generated and validated rabbit polyclonal antibodies [20, 21]. KLK9 was detected using a rabbit polyclonal antibody PAB-10236 (Orbigen, San Diego, CA). Species appropriate biotinylated secondary antibodies (Jackson ImmunoResearch Laboratories, West Grove, PA) followed by peroxidase-conjugated streptavidin (Dako, Carpinteria, CA) and standard 3',3'-diaminobenzadine tetrahydrochloride immunohistochemistry were used to visualize kallikrein specific immunoreactivity. All immunostained tissue sections were counterstained with Gills hematoxylin.

All stained sections were imaged with a Bliss digital imaging system pairing an Axioplan microscope (Zeiss, Jena, Germany) with a slide scanner (Bacus Laboratories, Center Valley, PA). Two independent observers completed scoring without knowledge of tumor demographics. Scoring parameters consisted of the staining intensity (range 1-3: low, medium and high) and the percent tumor core stained (range 1–4: 25 % increments). An IR score was calculated as the product of the staining intensity and percent stained. Scores from multiple cores from each tumor across the two observers were averaged [5]. Additional high-resolution images were prepared using a BX51 microscope (Olympus, Center Valley, PA) with a 100x-oil immersion objective and DP72 camera (Olympus).

### Statistical analysis

Kallikrein staining parameters in grade III and IV astrocytoma were compared using the Kruskal-Wallis test. Cumulative survival probabilities were estimated using the Kaplan-Meier method. Cox proportional hazards regression was used to assess the association of kallikrein staining parameters with survival in the grade 4 astrocytomas. Both univariable and multivariable analyses were completed including age, gender and ECOG performance score as covariates. Analysis of Martingale residuals from the Cox proportional hazards regression models were used to assess the functional form of kallikrein parameters. Appropriate cut points for kallikrein parameters were determined based on this analysis (22). The cut points established were utilized to create survival curves using the Kaplan-Meier method. The dichotomized kallikrein parameters were then assessed using Cox proportional hazards regression as described above. In all analyses, *P* < 0.05 was considered significant.

## Results

### Kallikrein protein expression is increased in grade IV astrocytoma

**Fig. 1:**
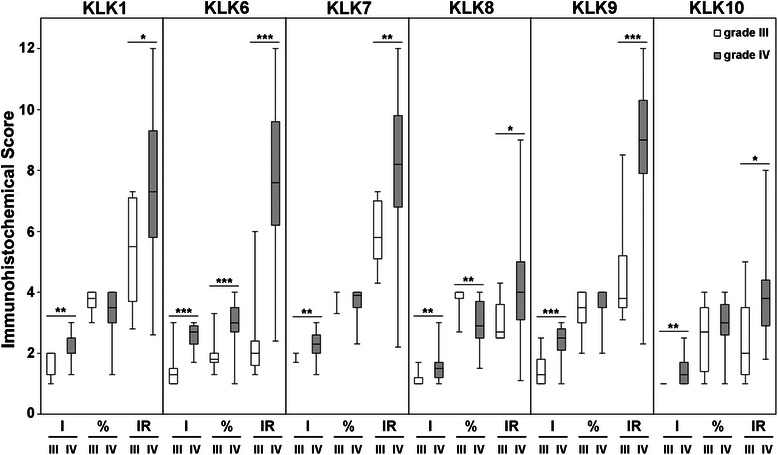
Immunohistochemical scores for KLK1, KLK6, KLK7, KLK8, KLK9 and KLK10 in grade IV versus grade III astrocytomas. Immunohistochemical scores for intensity (I), percent (%) and immunoreactivity score (IR) are shown as box and whisker plots. *P < 0.05, **P < 0.01 and ***P < 0.001; Kruskal-Wallis test. Mean and standard deviations are provided in Table 1

**Fig. 2:**
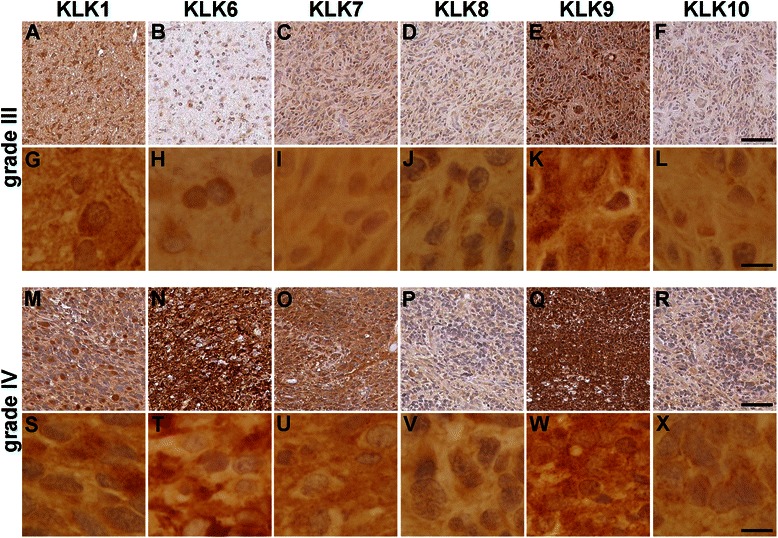
Immunohistochemical staining for KLK1, KLK6, KLK7, KLK8, KLK9 and KLK10 in astrocytomas. Representative photomicrographs of grade III astrocytomas (**a–l**) and grade IV astrocytomas (**m**–**x**) stained for KLK1 (**a**, **g**, **m** and **s**), KLK6 (**b**, **h, n** and **t**), KLK7 (**c, i, o** and **u**), KLK8 (**d, j, p** and **v**), KLK9 (**e, k, q** and **w**), or KLK10 (**f, l, r** and **x**). Images provided at low (**a–f** and **m–r**, scale bar = 50 μm) and high magnification (**g–l** and **s–x**, scale bar = 10 μm)

Expression of KLK1, KLK6, KLK7, KLK8, KLK9 and KLK10 protein was assessed by immunohistochemical analysis of grade III (*n* = 8) and grade IV (*n* = 60, *n* = 55 for KLK1) astrocytoma samples. The mean age was 48.1 (range 34–73) and 58.1 (range 33–84) for the grade III and grade IV patients, respectively. Grade III patients were 62.5 % female and grade IV patients were 36.7 % female. Additional demographic data regarding age, gender, extent of resection and ECOG performance scores are contained in Additional file 1: Table S1. All samples received a score for the intensity of the immunohistochemical staining, percent of the tissue core stained, and an IR score that is the product of the staining intensity and percent stained [5]. The range, median and distribution of scores are provided as box and whisker plots for each kallikrein by grade (Fig. 1). The most intense KLK-IR was seen in the grade IV tumors with antibodies recognizing KLK1, KLK6, KLK7 and KLK9. The intensity of immunoreactivity for KLK8 and KLK10 were low relative to the other kallikreins examined (see also Fig. 2).

To assess potential differences in kallikrein immunoreactivity across grade III and grade IV tumors, the mean scores for staining intensity, percent of tumor core stained, and IR scores were compared using the Kruskal-Wallis test (Table 1). The mean intensity and IR scores were significantly higher in grade IV compared to grade III for all kallikreins examined (Table 1; *P* < 0.03, Kruskal-Wallis test). Also, the mean percent tumor core stained was significantly higher in grade IV relative to grade III for KLK6 (Table 1; *P* = 0.0005, Kruskal-Wallis test). The mean percent tumor core stained was significantly higher in grade III compared to grade IV for KLK8 (Table 1; *P* = 0.0038, Kruskal-Wallis test). The mean percent tumor core stained for KLK1, KLK7, KLK9 or KLK10 across grades III and IV were not significantly different.

**Table 1:** Immunohistochemical scores for KLK1, KLK6, KLK7, KLK8, KLK9 and KLK10 in astrocytoma patients

	Intensity ± SD			Percent ± SD			IR ± SD		
	III	IV	*P* ^a^	III	IV	*P* ^a^	III	IV	*P* ^a^
KLK1	1.7 ± 0.4	2.2 ± 0.4	0.0063**	3.7 ± 0.4	3.3 ± 0.7	0.096	5.4 ± 1.8	7.3 ± 2.4	0.031*
KLK6	1.5 ± 0.7	2.6 ± 0.4	0.0003***	2.0 ± 0.6	3.0 ± 0.7	0.0005***	2.4 ± 1.5	7.7 ± 2.4	<0.0001***
KLK7	2.0 ± 0.1	2.3 ± 0.4	0.0042**	3.9 ± 0.2	3.7 ± 0.4	0.057	5.9 ± 1.1	8.2 ± 2.1	0.003**
KLK8	1.1 ± 0.2	1.5 ± 0.5	0.0099**	3.8 ± 0.5	3.0 ± 0.8	0.0038**	3.0 ± 0.7	4.3 ± 1.6	0.022*
KLK9	1.4 ± 0.6	2.4 ± 0.5	0.0003***	3.4 ± 0.7	3.6 ± 0.5	0.42	4.6 ± 1.8	9.0 ± 1.9	0.0001***
KLK10	1.0 ± 0.0	1.4 ± 0.4	0.0012**	2.5 ± 1.2	3.0 ± 0.7	0.36	2.4 ± 1.4	3.9 ± 1.5	0.011*

Mean and standard deviation of KLK1, KLK6, KLK7, KLK8, KLK9 and KLK10 staining intensity, percent tumor core stained and immunoreactivity score across 60 grade IV astrocytoma specimens

*P < 0.05, **P < 0.01 and ***P < 0.001

^a^Kruskal-Wallis test

The appearance of immunohistochemical staining for KLK1, KLK6, KLK7, KLK8, KLK9 and KLK10 in grade III and grade IV astrocytoma is shown in Fig. 2. As expected, immunoreactivity for each kallikrein was clearly visualized cytoplasmically. In addition, variable levels of extracellular staining were also apparent with the most intense extracellular staining seen in the case of KLK9, KLK7 and KLK6. In general, extracellular staining appeared the most intense in the higher grade gliomas (Fig. 2).

### Association of kallikrein immunoreactivity with survival in grade IV astrocytoma

To determine the potential impact of differential kallikrein protein expression on patient survival, univariable Cox proportional hazards regression analysis was applied to correlate kallikrein staining with patient survival. Previous analysis of this patient cohort demonstrated that elevated KLK6-IR was associated with decreased patient survival (hazard ratio [HR] = 1.16, 95 % confidence interval [CI] = 1.02–1.31, *P* = 0.02; Table 2) [5]. The current analysis additionally demonstrated that elevated KLK7-IR is associated with poor patient prognosis (HR = 1.17, CI = 1.02–1.34, *P* = 0.03; Table 2). Moreover, as the percent of the tumor core stained positive for KLK6 or KLK9 increased, survival decreased (HR = 1.63, CI = 1.06–2.50, *P* = 0.03 and HR = 2.25, CI = 1.21–4.22, *P* = 0.01, respectively; Table 2). After multivariable Cox modeling, utilizing gender, ECOG performance and age as covariates, the association of kallikreins with survival was no longer significant. However, when only gender and ECOG performance scores were included as covariates in the multivariable analysis, scores for KLK6-IR and KLK9 % tumor core stained, retained their significant association with survival (HR = 1.15, CI = 1.01–1.30, *P* = 0.03 and HR = 2.13, CI = 1.12–4.05, *P* = 0.021, respectively; Table 3).

**Table 2:** Astrocytoma survival association with kallikrein protein expression

	Univariable			Adjusted^a^		
	HR	95 % CI	*P*	HR	95 % CI	*P*
**KLK1**						
Intensity	0.93	0.49–1.78	0.83	1.10	0.54–2.23	0.80
Percent	1.21	0.81–1.80	0.36	1.41	0.92–2.15	0.11
IR	1.08	0.96–1.21	0.21	1.13	0.99–1.29	0.06
**KLK6**						
Intensity	0.89	0.42–1.90	0.77	0.73	0.32–1.68	0.46
Percent	1.63	1.06–2.50	0.03*	1.35	0.87–2.10	0.18
IR	1.16	1.02–1.31	0.02*	1.10	0.96–1.25	0.17
**KLK7**						
Intensity	1.25	0.61–2.56	0.54	0.95	0.45–2.03	0.90
Percent	1.96	0.91–4.22	0.09	1.91	0.87–4.19	0.11
IR	1.17	1.02–1.34	0.03*	1.10	0.95–1.28	0.20
**KLK8**						
Intensity	0.87	0.46–1.66	0.68	0.84	0.40–1.78	0.65
Percent	0.91	0.66–1.25	0.54	0.96	0.68–1.36	0.81
IR	0.98	0.83–1.16	0.81	0.95	0.77–1.17	0.61
**KLK9**						
Intensity	0.65	0.38–1.13	0.13	0.64	0.36–1.14	0.13
Percent	2.25	1.21–4.22	0.01*	1.68	0.86–3.30	0.13
IR	1.05	0.90–1.22	0.52	0.99	0.85–1.16	0.90
**KLK10**						
Intensity	1.14	0.59–2.20	0.70	0.99	0.46–2.12	0.97
Percent	0.99	0.66–1.48	0.94	1.10	0.72–1.69	0.67
IR	1.12	0.92–1.37	0.27	1.03	0.82–1.28	0.80

Cox proportional hazards regression analysis assessing the association of kallikrein protein expression in 60 grade IV astrocytoma specimens. *P ≤ 0.03, Cox proportional hazards regression analysis

^a^Adjusted for age, gender and performance score

**Table 3:** Survival analysis of immunohistochemical scores after cut point analysis

			Univariable Analysis			Multivariable Analysis		
	*N*	Median Survival	Hazard Ratio	95 % CI	*P* ^a^	Hazard Ratio	95 % CI	*P* ^a^
**KLK6 %**	60	355	1.63	1.06–2.50	0.027	1.35	0.87–2.10	0.18^b^
						1.55	1.10–2.40	0.052^c^
<3	32	419						
≥3	28	320	2.07	1.14–3.76	0.017	1.36	0.72–2.57	0.34^b^
						1.93	1.03–3.63	0.04^c^
**KLK6-IR**	60	355	1.16	1.02–1.31	0.020	1.10	0.96–1.25	0.17^b^
						1.15	1.01–1.30	0.03^c^
<10	48	408						
≥10	12	276	2.36	1.19–4.68	0.014	1.72	0.84–3.54	0.14^b^
						2.4	1.20–4.78	0.01^c^
**KLK7-IR**	60	355	1.17	1.02–1.34	0.025	1.10	0.95–1.28	0.20^b^
						1.15	1.00–1.32	0.057^c^
<9	38	422						
≥9	22	294	2.15	1.20–3.87	0.010	1.47	0.77–2.80	0.24^b^
						2.04	1.10–3.77	0.024^c^
**KLK9 %**	60	355	2.25	1.21–4.22	0.011	1.68	0.86–3.3	0.13^b^
						2.13	1.12–4.05	0.021^c^
<4	29	422						
=4	31	311	1.70	0.98–2.95	0.061	1.42	0.79–2.57	0.24^b^
						1.58	0.89–2.79	0.12^c^

Cox proportional hazards regression analysis assessing the association of kallikrein protein expression with patient survival in 60 grade IV astrocytoma specimens

^a^Proportional hazards regression

^b^Adjusted for age, gender and performance score

^c^Adjusted for gender and performance score

**Fig. 3:**
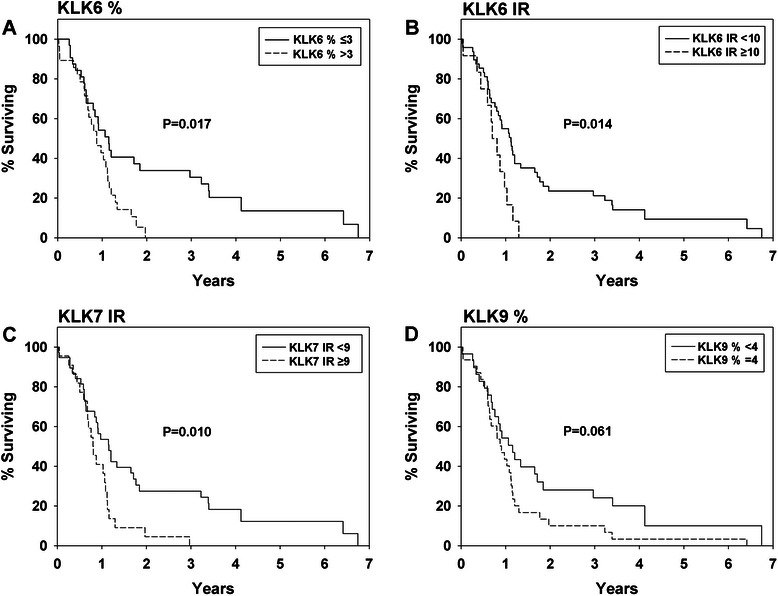
Kaplan-Meier curves for overall survival of grade IV astrocytoma patients Curves are shown for (**a**) KLK6 % of tumor core stained of ≤ 3 or > 3, (**b**) KLK6-IR scores of < 10 or ≥ 10, (**c**) KLK7-IR scores of < 9 or ≥ 9, and (**d**) KLK9 % of tumor core stained of < 4 or = 4. *P* values shown are based on proportional hazards regression

Martingale residuals from the Cox proportional hazards regression model were used to assess the functional form of kallikrein parameters and when appropriate dichotomize the patients into groups. As we recently reported, patients with a KLK6-IR < 10 (*n* = 48) have a median survival of 408 d, while patients with KLK6-IR ≥ 10 (*n* = 12) have a significantly shorter survival time, 276 d (HR = 2.36, CI = 1.19–4.68, *P* = 0.014; Table 3 and Fig. 3) [5]. This comparison remained significant when the data were adjusted for gender and ECOG performance scores (HR = 2.4, CI = 1.20–4.78, *P* = 0.01; Table 3). Patients with KLK6 % < 3 (*n* = 32) had a median survival of 419 d; patients with KLK6 % ≥ 3 (*n* = 28) had a significantly shorter median survival of 320 d (HR = 2.07, CI = 1.14–3.76, *P* = 0.017; Table 3 and Fig. 3). Multivariable analysis of KLK6 % tumor core stained showed a significant correlation with survival, utilizing gender and ECOG as covariates (HR = 1.93, CI = 1.03–3.63, *P* = 0.04; Table 3). Patients with KLK7-IR < 9 (*n* = 38) had a median survival of 422 d; patients with KLK7-IR ≥ 9 (*n* = 22) had a significantly shorter median survival of 294 d (HR = 2.15, CI = 1.20–3.87, *P* = 0.01), which remained significantly different after adjustment for gender and performance score (HR = 2.04, CI = 1.10–3.77, *P* = 0.024). Patients with KLK9 % tumor core stained < 4 (*n* = 29) had median survival of 422 d; patients with KLK9 % tumor core stained = 4 (*n* = 31) had a median survival of 311 d (HR = 1.70, CI = 0.98–2.95, *P* = 0.061; Table 3 and Fig. 3).

## Discussion

Through a comprehensive parallel analysis of immunoreactivity for six kallikreins in grade III and IV astrocytoma we determined that tumor core staining for KLK7 and KLK9, like KLK6, has significant prognostic value with regard to patient survival. Specifically, higher tumor core levels of KLK6, KLK7 and KLK9 were each associated with reduced GBM patient survival. These findings highlight the likely significance of each of these secreted serine proteases to the pathophysiology of high-grade glioma and the need for additional studies to define the mechanism of action, significance to therapy resistance and their potential utility as therapeutic targets to improve patient outcomes.

Prior studies demonstrate that higher levels of KLK6 immunoreactivity in grade IV astrocytoma are associated with poor patient survival [5]. Expression of KLK6 RNA is also linked to poor patient survival in a group of intracranial malignancies, including glioblastomas, meningiomas, oligodendrogliomas, ependymomas and other rare malignancies and brain metastases [12]. KLK6 expression is associated with an unfavorable prognosis in ovarian [3] and colorectal cancers [22]. In contrast, KLK6 expression in breast cancer appears to have a protective role, with elevated expression being linked to reduced proliferation and tumorigenicity. Expression of KLK6 is also reduced in metastatic lymph nodes [23].

Paralleling what we have reported for KLK6 [5], results herein suggest that elevated levels of KLK7 expression are associated with poor patient survival. Supporting this, a prior study examining a mixed cohort of intracranial malignancies similarly demonstrated that elevations in KLK7 expression are associated with poor patient survival [13]. Both studies support the idea that KLK7 expression leads to more aggressive brain tumors. KLK7 is also associated with reduced survival times in ovarian [24], breast [25] and colon cancer [26]. By contrast, the current study and prior efforts suggest that changes in KLK8 expression in GBM or other intracranial malignancies have little association with prognosis [13]. In head and neck squamous cell carcinoma, KLK8 expression is down regulated in metastases, but it is not associated with improved survival [27]. Pointing to potential cancer-type specific effects as described for KLK6, KLK8 is a favorable prognostic indicator in ovarian cancer [28], but an unfavorable indicator in lung cancer [29].

The current study provides the first evidence that KLK9 protein expression may be associated with poor prognosis in glioma patients. Again pointing to potential tumor specific effects of kallikreins, KLK9 was previously found to be expressed at higher levels in low grade breast and ovarian cancers [30, 31]. In breast cancer, KLK9 expression was also higher in patients with smaller tumors and was associated with increased patient survival, particularly patients that are estrogen and progesterone receptor negative [31]. In the case of ovarian cancer, KLK9 expression is a predictor of longer overall survival in patients with lower grade tumors [30]. Specific regulatory patterns of kallikreins across cancers may be related to hormone regulation, as proposed for KLK9 in both breast and ovarian cancer. Interestingly, KLK10 is a proposed tumor suppressor gene in breast carcinoma cell lines, blocking tumorigenicity in an *in vivo* breast cancer model [32]. However, underscoring the importance of considering tumor-type specific effects, KLK10 has been found to be an unfavorable prognostic indicator in gastric, colorectal and ovarian cancer [33–35]. Here we report that KLK10 expression is increased with astrocytoma grade, but no association with patient survival was observed.

Our previous studies demonstrate that KLK6 over expression promotes resistance of GBM cell lines to cell death inducing agents, including radiation and temozolomide, the current standard of GBM patient care [5]. Interestingly, the KLK6-mediated activation of PAR1 was shown to play an essential role in its ability to promote glioma cell survival [5]. Notably, KLK6 also promotes survival of the Jurkat leukemia T cell line in a PAR1 dependent fashion [36]. In melanoma cells, PAR1 is activated by KLK6 promoting intracellular calcium flux and tumor cell invasion [37]. Together these studies support the concept that KLK6 mediates its effects in astrocytoma cells in a PAR1-dependent fashion and that this is likely to involve both cell survival and invasion.

KLK7 overexpression increases cell invasion in glioma cell lines *in vitro* matrigel assay [13]. Moreover, overexpression of KLK7 in colon cancer cell lines promotes proliferation and tumorigenicity [38]. The signaling pathways participating in KLK7-mediated effects in tumor cells have not been elucidated, but effects on cell invasion and proliferation may account for the shortened survival times we observe in GBM patients with elevated tumor KLK7 expression.

The current studies are among the first to examine KLK9 in malignancy and results of interest since elevations in tumor KLK9 were found to be associated with higher grade gliomas. These findings point to the need for additional studies to determine the biological effects of kallikreins in glioma malignancy and whether these effects are mediated by PAR-dependent and/or independent actions, such as extracellular matrix turnover. In addition to direct effects on tumor cell behavior, it is also possible that the kallikreins identified herein participate in activation, or inactivation, cascades with other kallikreins or enzymes involved in the fibrinolytic or thrombolytic systems [21].

## Conclusion

Data presented here demonstrate that elevated levels of KLK6, KLK7 and KLK9 proteins are associated with poor GBM patient survival. Our prior studies suggest that KLK6 directly promotes glioma cell survival, including resistance to radiation and temozolomide, in a PAR1-dependent manner. The current work therefore points to the need for additional studies to determine the potential pathophysiological roles of KLK7 and KLK9 in glioma malignancy and any parallel involvement of PAR-activation in mediating their effects. This knowledge will be key to potential future studies in which kallikreins or the receptors they activate could be targeted therapeutically to improve patient survival. Importantly, the current results suggest future studies to determine any impact of elevated kallikrein levels on therapy responsiveness. Finally, analysis of the mechanisms by which kallikreins are elevated in GBM, albeit by gene duplications, hormonal regulation, epigenetic changes, or other means, will be of interest and potentially important to understanding the differential expression and outcomes these novel serine proteases exert across a wide range of malignancies.

## Additional files

Additional file 1: Table S1. Patient demographic data. (DOCX 40 kb)

## Abbreviations

KLK: Kallikrein
IR: Immunoreactivity score
PSA: Prostate specific antigen
GBM: Glioblastoma multiforme
CNS: Central nervous system
WHO: World Health Organization
ECOG: Eastern Cooperative Oncology Group
HR: Hazard ratio
CI: Confidence interval
