# Supplementary material for: Prognostic significance of multiple kallikreins in high-grade astrocytoma
Source: BMC Cancer. 2015 Aug 1;15:565. doi: 10.1186/s12885-015-1566-5 (PMC4521496; doi:10.1186/s12885-015-1566-5)
Supplement: Additional file 1: Table S1. — Patient demographic data. (DOCX 40 kb) [file 12885_2015_1566_MOESM1_ESM.docx]

|  | **Grade 3** |  | **Grade 4** |
| --- | --- | --- | --- |
| N | 8 |  | 60 |
| Age at surgery, Mean ± SD | 48.1 ± 13.0 |  | 58.1 ± 13.0 |
| Age range | 34-73 |  | 33-84 |
| Female, n (%) | 5 (62.5) |  | 22 (36.7) |
| Gross total resection, n (%) | 3 (37.5) |  | 31 (51.7) |
| Performance score* ≥2, n (%) | 3 (37.5) |  | 22 (36.7) |

* Eastern Cooperative Oncology Group (ECOG) performance score.
